# Dataset of the electrochemical potential windows for the Au(*hkl*)|ionic liquid interfaces defined by the cut-off current densities

**DOI:** 10.1016/j.dib.2021.107585

**Published:** 2021-11-18

**Authors:** Hiroyuki Ueda, Soichiro Yoshimoto

**Affiliations:** a Graduate School of Science and Technology, Kumamoto University, 2-39-1 Kurokami, Chuo-ku, Kumamoto 860-8555, Japan; b Institute of Industrial Nanomaterials, Kumamoto University, 2-39-1 Kurokami, Chuo-ku, Kumamoto 860-8555, Japan

**Keywords:** Au(*hkl*), Electrical double layer, Electrochemical potential window, Interfacial processes, Ionic liquids, Linear sweep voltammetry, Single crystal electrodes, Specific desorption

## Abstract

This data article describes the linear sweep voltammetry (LSV) profiles of five ionic liquids (ILs) at the low-index (*hkl*) (*hkl* = 111, 100, and 110) planes of Au. The LSV profiles were recorded at 25 ± 1°C for the Au(*hkl*)|IL interfaces maintained in a hanging meniscus configuration in an inert Ar atmosphere (with H_2_O and O_2_ concentrations being lower than 5 ppm). The width of the electrical double-layer regions (*E*_dl_) and the electrochemical potential windows (*E*_pw_) of the ILs were evaluated based on the cut-off current densities (*j*_cut-off_): ±5, ±10, and ±20 µA cm^–2^ for *E*_dl_ and ±0.1, ±0.5, and ±1.0 mA cm^–2^ for *E*_pw_. The potential values were calibrated to the redox potential of ferrocene/ferrocenium in each IL. A detailed discussion on the electrochemical behaviors of the ILs on Au(*hkl*) is provided in the related article “Voltammetric Investigation of Anodic and Cathodic Processes at Au(*hkl*)|Ionic Liquid Interfaces”, published in the Journal of Electroanalytical Chemistry (Ueda and Yoshimoto, 2021).

## Graphical abstract

**Figure:**
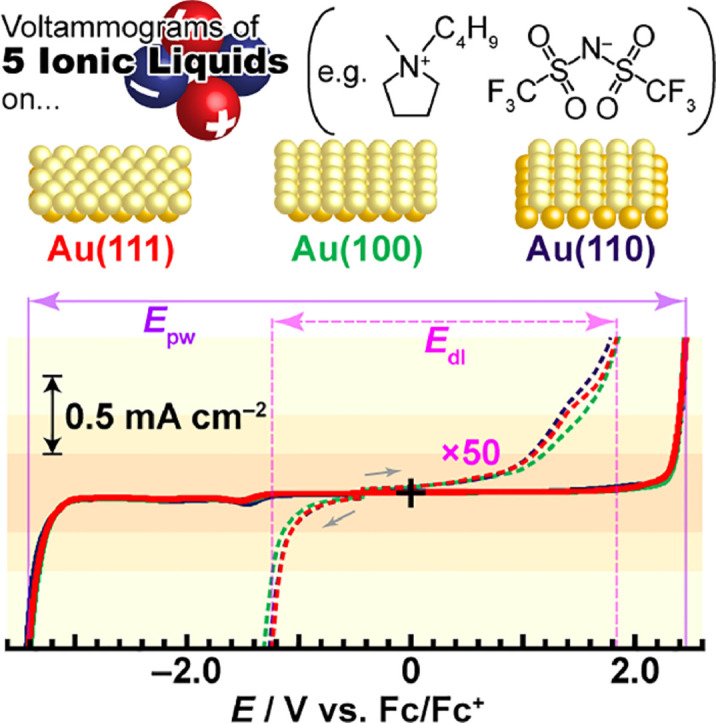

## Specifications Table

**Table:** 

Subject	Electrochemistry
Specific subject area	Surface electrochemistry of ionic liquids (ILs)
Type of data	Table Graph
How the data were acquired	A CH Instruments potentiostat (Model 610D) was used for linear sweep voltammetry (LSV). The scan rate was 50 mV s^–1^. LSV was performed for Au(*hkl*) working electrodes (*hkl* = 111, 100, and 110) contacted with vacuum-dried ILs at 25 ± 1°C in three-electrode cells with Pt wires as counter and quasi-reference electrodes. Electrochemical measurements for each electrode were conducted in an Ar atmosphere (H_2_O and O_2_ <5 ppm) in four steps: the electrode potential was (1) swept to the positive direction until the current density reached 20 µA cm^–2^, (2) swept to the negative direction until the current density reached –20 µA cm^–2^, (3) swept to the positive direction until the current density reached 1 mA cm^–2^, and (4) swept to the negative direction until the current density reached –1 mA cm^–2^. Prior to LSV, the electrode was maintained at –0.1 V vs. Pt during the holding time of 10 s for (1) and 2 min for (2), (3), and (4). Voltammograms obtained via (1) and (2) were used to evaluate the widths of the electrical double-layer region (*E*_dl_), while those obtained via (3) and (4) were used to determine the electrochemical potential windows (*E*_pw_).
Data format	Raw Analyzed
Description of data collection	Raw LSV data were exported to Microsoft Excel to plot the voltammograms and analyze the *E*_dl_ and *E*_pw_ of ILs on Au(*hkl*). The cut-off current densities (*j*_cut-off_) for *E*_dl_ were ±5, ±10, and ±20 µA cm^–2^, whereas *j*_cut-off_ for *E*_pw_ were ±0.1, ±0.5, and ±1.0 mA cm^–2^. The anodic and cathodic limits of *E*_dl_ and *E*_pw_ were determined based on the *j*_cut-off_ values.
Data source location	• *Institution: Kumamoto University* • *City/Town/Region: Kumamoto* • *Country: Japan* • *Latitude and longitude (and GPS coordinates, if possible) for collected samples/data: 32.81291, 130.72578*
Data accessibility	Repository name: Mendeley Data Data identification number (DOI): http://doi.org/10.17632/tv4cm845wv.1[2] Direct URL to data: http://doi.org/10.17632/tv4cm845wv.1
Related research article	[1] H. Ueda, S. Yoshimoto, Voltammetric Investigation of Anodic and Cathodic Processes at Au(*hkl*)|Ionic Liquid Interfaces, *J. Electroanal. Chem.* 900 (2021) 115691.

## Value of the Data

• The electrochemical data reported herein are valuable because they can provide fundamental information on Au(*hkl*)|IL interfaces for electrochemical studies.
• Electrochemists can benefit from these data because it will aid them in selecting appropriate potential ranges for studies using Au(*hkl*)|IL interfaces. For instance, the decomposition of ILs can be significantly reduced by limiting the potential of the Au(*hkl*) working electrode to within the *E*_dl_*.*
• These data can be used to gain further insights into the origin of each anodic or cathodic process occurring at Au(*hkl*)|IL interfaces by means of microscopic or spectroscopic techniques such as scanning tunneling microscopy [3], [4], [5] and differential electrochemical mass spectroscopy [6].
• These data were analyzed at different *j*_cut-off_ values, thereby providing a basis for a fair comparison of *E*_dl_ and *E*_pw_ between different electrode|IL interfaces.
• In addition, these data are useful for identifying potential regions in which ILs exhibit nearly ideal capacitive behavior. Such potential regions are essential for ensuring the accuracy of microcalorimetric measurements [7] and amperometric sensors using ILs [8].

## 1 Data Description

**Fig. 1:**
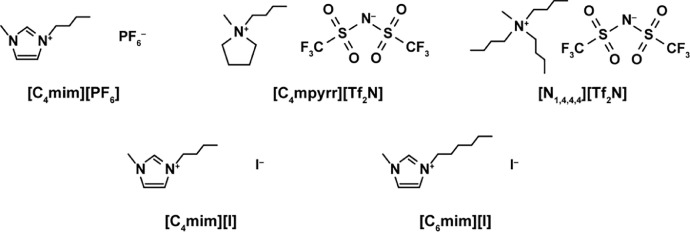
The chemical structures of the ILs.

This data article summarizes the LSV profiles, *E*_dl_, and *E*_pw_ of the Au(*hkl*)|IL interfaces. The chemical structures of the five ILs are shown in Fig. 1. The raw data of all LSV profiles and Tables can be found in the repository (see “Data accessibility” in the Specifications Table) [2].

**Fig. 2:**
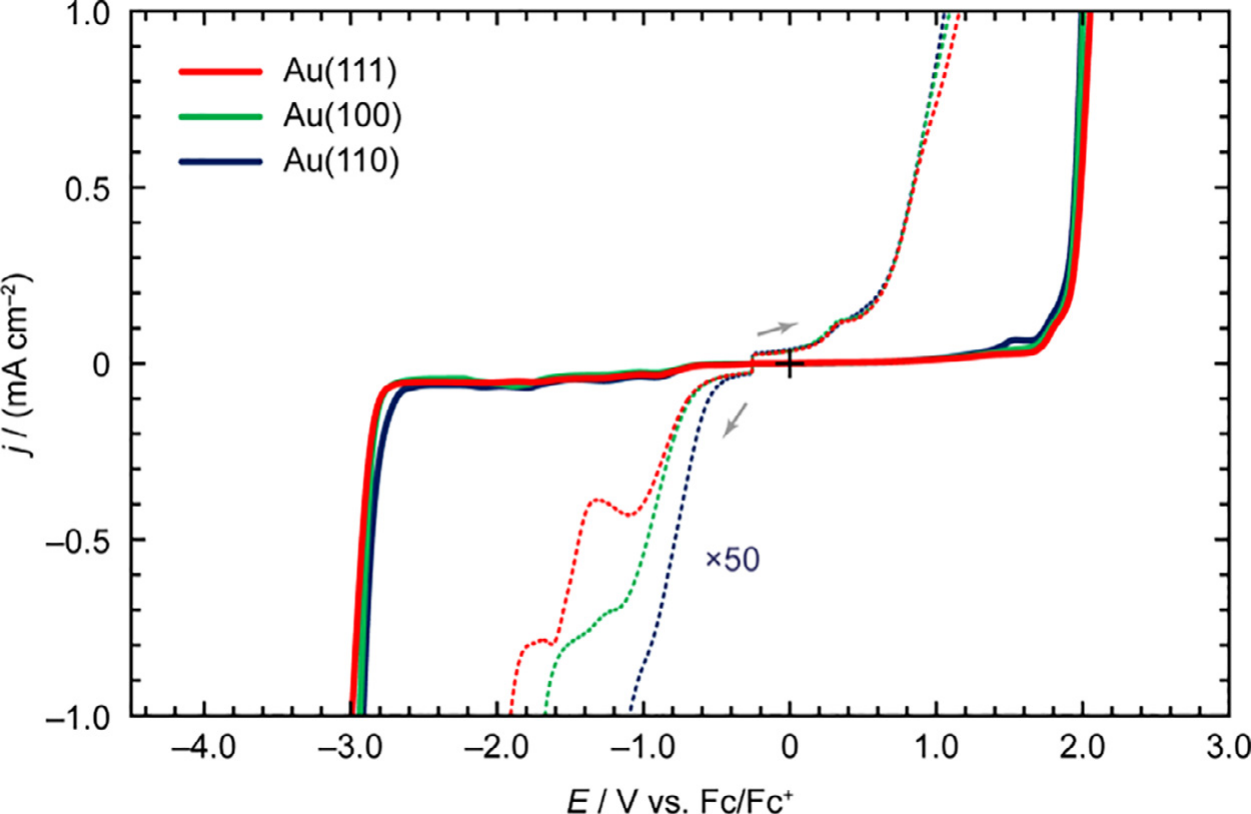
LSV profiles of [C_4_mim][PF_6_] on Au(*hkl*) recorded at the scan rate of 50 mV s^–1^.

Fig. 2 shows the LSV profiles of 1-butyl-3-methylimidazolium hexafluorophosphate ([C_4_mim][PF_6_]) on Au(*hkl*). In the enlarged voltammograms (the dotted lines), two reductive peaks at –1.11 and –1.63 V vs. Fc/Fc^+^ appeared for the Au(111) surface, whereas the reductive peaks were unclear for Au(100) and Au(110). As shown using the solid lines, the oxidation onset potential of [C_4_mim][PF_6_] on Au(110) was more negative than that on Au(111) and Au(100). Similarly, the reduction onset potential of [C_4_mim][PF_6_] on Au(110) was more positive than that on the other crystal faces. In addition, an oxidation process was observed prior to a massive increase in the oxidation current density of [C_4_mim][PF_6_] on Au(110).

**Fig. 3:**
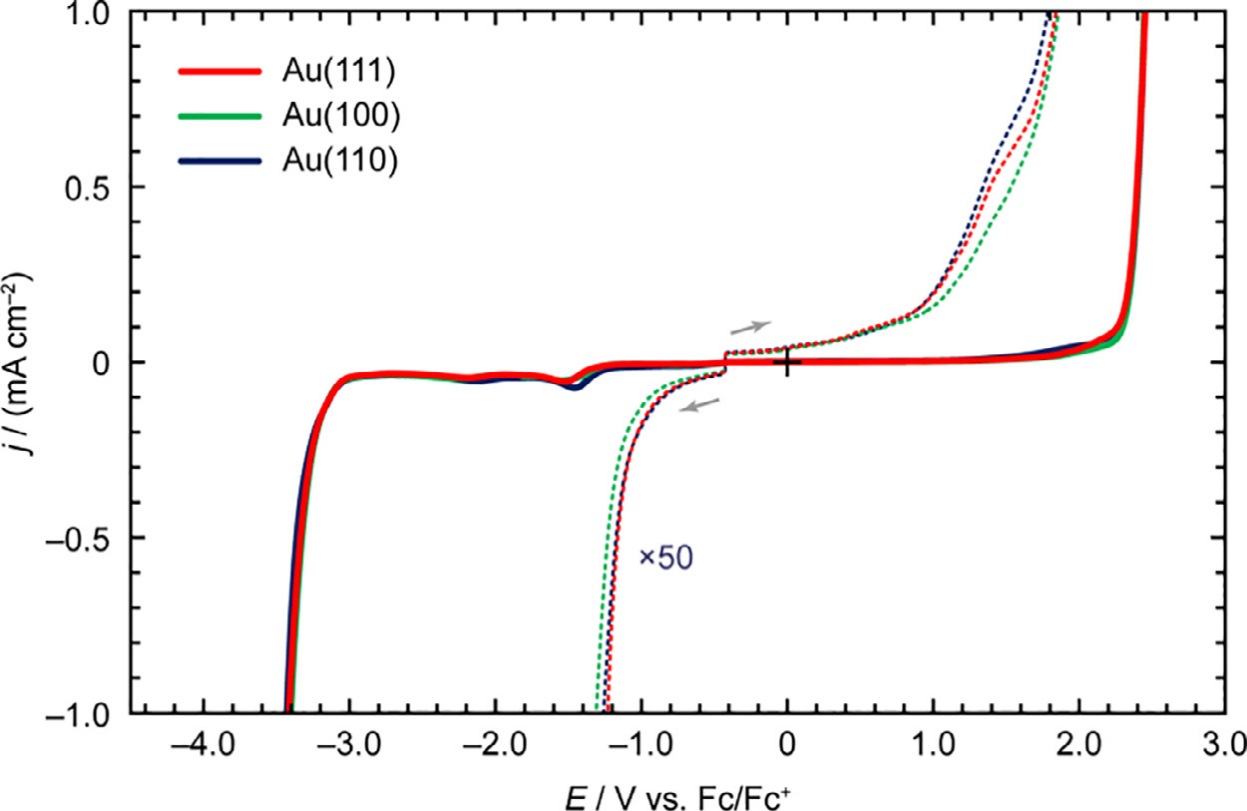
LSV profiles of [C_4_mpyrr][Tf_2_N] on Au(*hkl*) recorded at the scan rate of 50 mV s^–1^.

Fig. 3 depicts the LSV profiles of *N*-butyl-*N*-methylpyrrolidinium bis(trifluoromet-hylsulfonyl)amide ([C_4_mpyrr][Tf_2_N]) on Au(*hkl*). The enlarged voltammogram at the anodic scan of [C_4_mpyrr][Tf_2_N] on Au(111) was nearly identical to that of Au(110), except for the presence of a shoulder peak at approximately 1.40 V vs. Fc/Fc^+^. Furthermore, both crystal faces generated nearly identical voltammetric shapes during the cathodic scan prior to reaching –20 µA cm^–2^. Conversely, in the *E*_dl_ region, the anodic and cathodic processes on Au(100) were milder than those on the other crystal faces. In the voltammograms recorded to determine the *E*_pw_ (the solid lines), the voltammetric shape between 1.20 V vs. Fc/Fc^+^ and *E*_pw-AL_ and the peak position and peak current density of the cathodic process at around –1.50 V vs. Fc/Fc^+^ were dependent on the crystallographic orientation of gold.

**Fig. 4:**
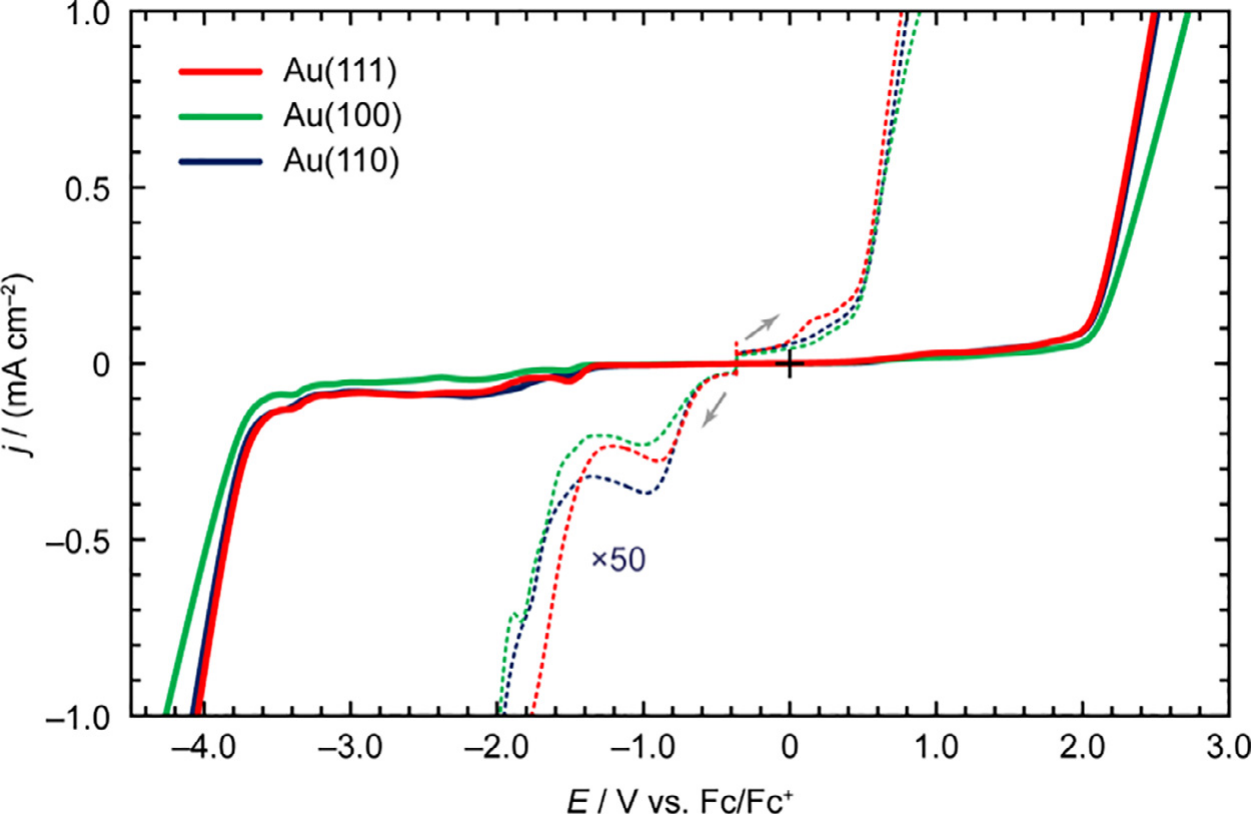
LSV profiles of [N_1,4,4,4_][Tf_2_N] on Au(*hkl*) recorded at the scan rate of 50 mV s^–1^.

Fig. 4 shows the LSV profiles of tributylmethylammonium bis(trifluoromethylsulfonyl)amide ([N_1,4,4,4_][Tf_2_N]) on Au(*hkl*). In the *E*_dl_ region (indicated using the dotted lines), a small anodic process was observed at approximately 0.1 V vs. Fc/Fc^+^ solely for the Au(111) surface. All crystal faces exhibited a cathodic peak at approximately –1.00 V vs. Fc/Fc^+^. The absolute value of the peak current density for this cathodic process was evaluated to be in the following order: Au(100) < Au(111) < Au(110). In the *E*_pw_ region (indicated using the solid lines), the voltammetric shapes of [N_1,4,4,4_][Tf_2_N] on Au(111) and Au(110) were nearly identical, except for the difference in the peak current density of the cathodic process at approximately –1.50 V vs. Fc/Fc^+^. In contrast, the absolute value of the current density (|*j*|) for Au(100) tended to be the lowest over the entire potential range.

**Fig. 5:**
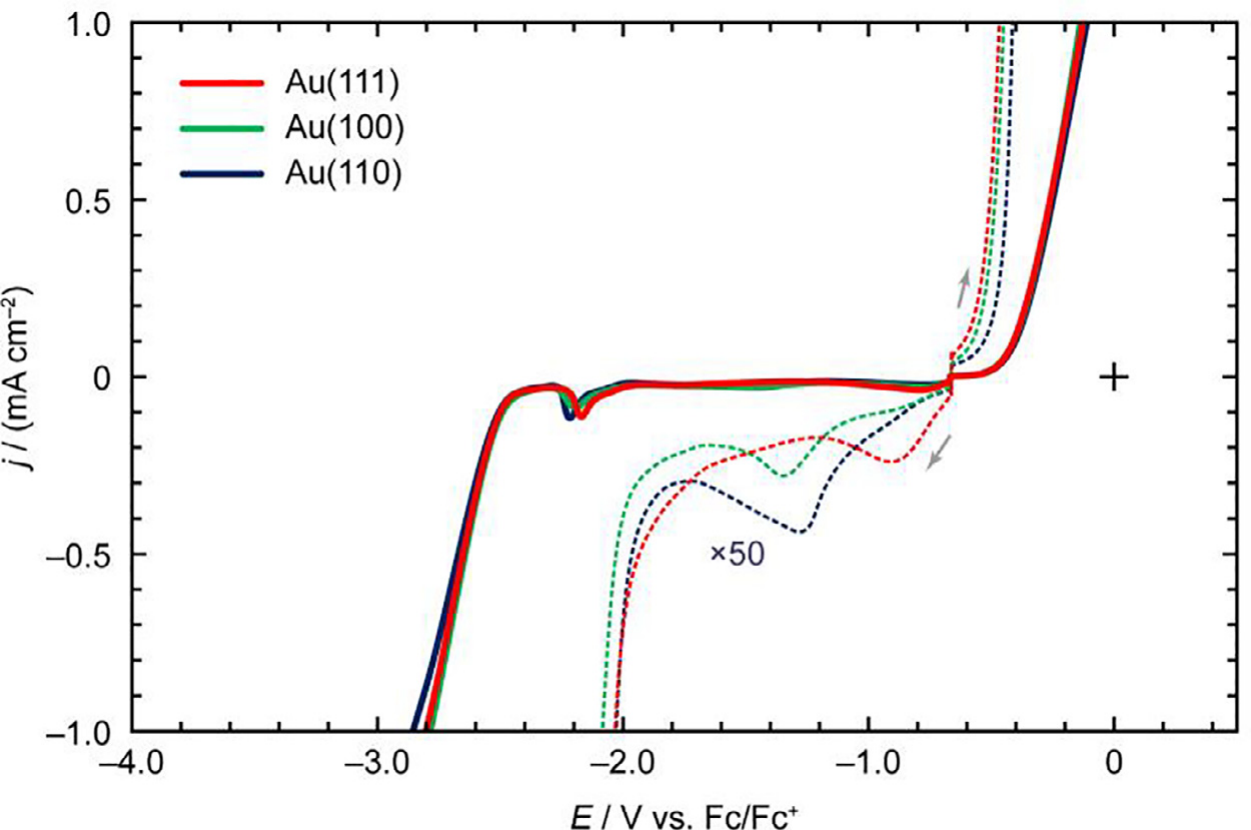
LSV profiles of [C_4_mim][I] on Au(*hkl*) recorded at the scan rate of 50 mV s^–1^.

Fig. 5 illustrates the LSV profiles of 1-butyl-3-methylimidazolium iodide ([C_4_mim][I]) on Au(*hkl*). As indicated using the dotted lines, the order of the onset oxidation potential was evaluated as Au(111) < Au(100) < Au(110). The cathodic peak potentials were –0.92 V vs. Fc/Fc^+^ for Au(111), –1.35 V vs. Fc/Fc^+^ for Au(100), and –1.29 V vs. Fc/Fc^+^ for Au(110). As indicated using the solid lines, no significant differences in the voltammetric shape at the anodic scan were identified between Au(*hkl*). During the cathodic scan, a voltammetric peak generated by the from the reductive desorption of the iodine adlayer on Au(*hkl*) appeared at approximately –2.20 V vs. Fc/Fc^+^ [1], [9]. Furthermore, the |*j*| value during the *E*_pw-CL_ determining reduction was lowest for Au(110).

**Fig. 6:**
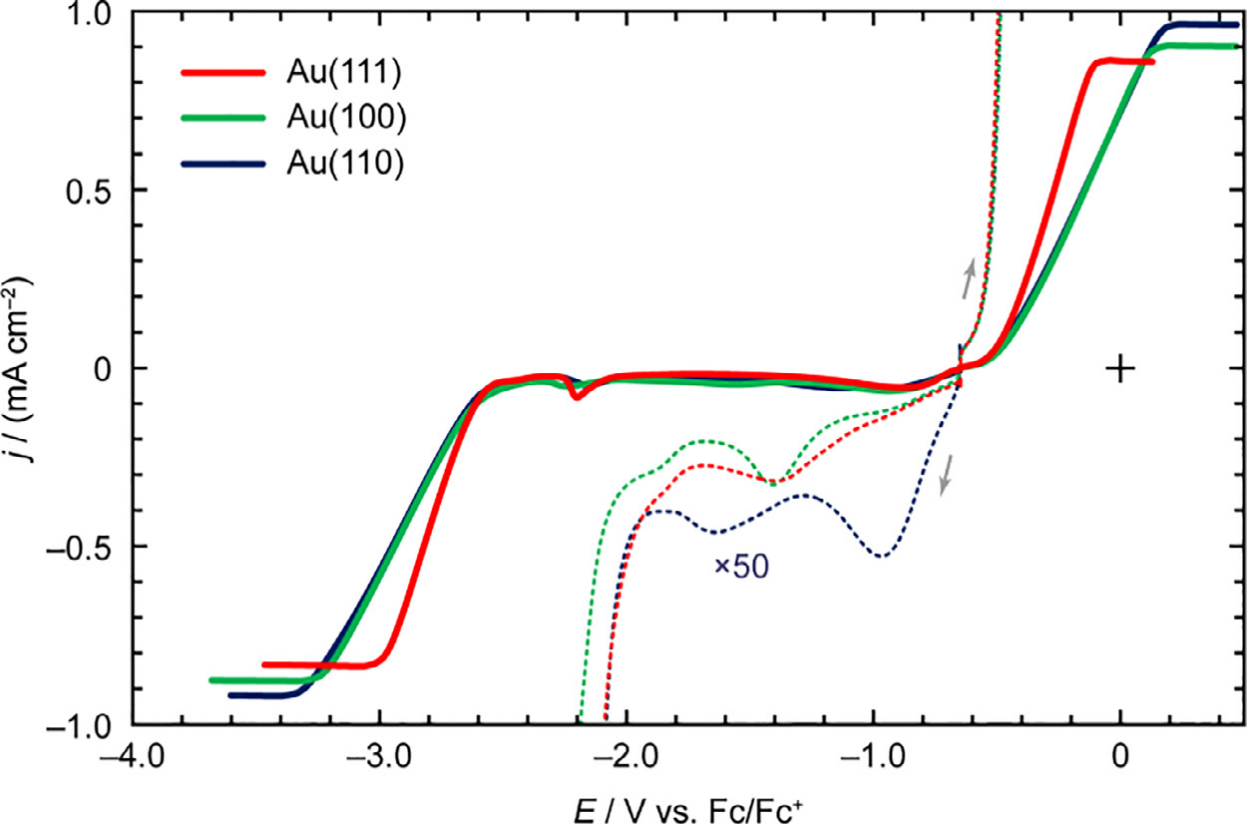
LSV profiles of [C_6_mim][I] on Au(*hkl*) recorded at the scan rate of 50 mV s^–1^.

Fig. 6 shows the LSV profiles of 1-hexyl-3-methylimidazolium iodide ([C_6_mim][I]) on Au(*hkl*). The enlarged voltammograms of Au(*hkl*) in the anodic scan were nearly identical. The cathodic peak appeared at –1.40 V vs. Fc/Fc^+^ for Au(111) and Au(100). As for the Au(110) surface, the two cathodic peaks were observed at –0.97 and –1.64 V vs. Fc/Fc^+^. In the *E*_pw_ region (indicated using the solid lines), all the voltammograms exhibited the maximum |*j*| values during the anodic and cathodic scans, which were between 0.5 and 1.0 mA cm^–2^ and in the following order: Au(111) < Au(100) < Au(110). The voltammetric shape for the reductive desorption of the iodine adlayer at approximately –2.20 V vs. Fc/Fc^+^ was dependent on the crystallographic orientation of gold.

Tables 1 and 2 summarize the *E*_dl_ and *E*_pw_ of [C_4_mim][PF_6_] on Au(*hkl*), respectively. The *E*_dl-AL_ of [C_4_mim][PF_6_] was estimated to be Au(110) < Au(100) < Au(111) and *E*_dl-CL_ for [C_4_mim][PF_6_] followed the order: Au(111) < Au(100) < Au(110). Therefore, the *E*_dl_ value of [C_4_mim][PF_6_] was evaluated as Au(110) < Au(100) < Au(111). Similarly, the *E*_pw-AL_ of [C_4_mim][PF_6_] followed the order: Au(110) < Au(100) < Au(111), and the *E*_pw-CL_ of [C_4_mim][PF_6_] was regarded as Au(111) < Au(100) < Au(110), suggesting that the electrochemical stability of [C_4_mim][PF_6_] on the electrode surface followed the order: Au(110) < Au(100) < Au(111).

**Table 1:** The *E*_dl-AL_, *E*_dl-CL_, and *E*_dl_ of [C_4_mim][PF_6_] on Au(*hkl*) at different *j*_cut-off_ values.

*j*_cut-off_/±µA cm^–2^	Crystal face	*E*_dl-CL_/V vs. Fc/Fc^+^	*E*_dl-AL_/V vs. Fc/Fc^+^	*E*_dl_/V
5	Au(111)	–0.86	0.68	1.53
	Au(100)	–0.82	0.68	1.50
	Au(110)	–0.66	0.67	1.33

10	Au(111)	–1.44	0.84	2.28
	Au(100)	–0.98	0.83	1.81
	Au(110)	–0.79	0.83	1.62

20	Au(111)	–1.91	1.15	3.06
	Au(100)	–1.68	1.09	2.76
	Au(110)	–1.10	1.05	2.15

**Table 2:** The *E*_pw-AL_, *E*_pw-CL_, and *E*_pw_ of [C_4_mim][PF_6_] on Au(*hkl*) at different *j*_cut-off_ values.

*j*_cut-off_/±mA cm^–2^	Crystal face	*E*_pw-CL_/V vs. Fc/Fc^+^	*E*_pw-AL_/V vs. Fc/Fc^+^	*E*_pw_/V
0.1	Au(111)	–2.80	1.80	4.60
	Au(100)	–2.79	1.78	4.57
	Au(110)	–2.69	1.75	4.44

0.5	Au(111)	–2.92	1.99	4.91
	Au(100)	–2.89	1.96	4.85
	Au(110)	–2.86	1.95	4.81

1.0	Au(111)	–2.99	2.05	5.05
	Au(100)	–2.94	2.01	4.94
	Au(110)	–2.92	2.00	4.91

Tables 3 and 4 list the *E*_dl_ and *E*_pw_ of [C_4_mpyrr][Tf_2_N] on Au(*hkl*). Regardless of *j*_cut-off_, the *E*_dl-AL_ for [C_4_mpyrr][Tf_2_N] followed the order: Au(110) < Au(111) < Au(100), and the *E*_dl-CL_ for [C_4_mpyrr][Tf_2_N] was evaluated as Au(100) < Au(110) < Au(111). Therefore, the most electrochemically stable crystal face for [C_4_mpyrr][Tf_2_N] in the EDL region was estimated to be Au(100), whereas a relatively narrower *E*_dl_ of [C_4_mpyrr][Tf_2_N] was obtained for the Au(110) and Au(111) surfaces. Conversely, the *E*_pw_ values (e.g., 5.85‒5.88 V at *j*_cut-off_ = ±1.0 mA cm^–2^) were almost the same for all the gold single crystal electrodes.

**Table 3:** The *E*_dl-AL_, *E*_dl-CL_, and *E*_dl_ of [C_4_mpyrr][Tf_2_N] on Au(*hkl*) at different *j*_cut-off_ values.

*j*_cut-off_/±µA cm^–2^	Crystal face	*E*_dl-CL_/V vs. Fc/Fc^+^	*E*_dl-AL_/V vs. Fc/Fc^+^	*E*_dl_/V
5	Au(111)	–1.07	1.09	2.16
	Au(100)	–1.14	1.19	2.33
	Au(110)	–1.07	1.07	2.15

10	Au(111)	–1.17	1.38	2.55
	Au(100)	–1.23	1.53	2.76
	Au(110)	–1.18	1.33	2.51

20	Au(111)	–1.23	1.84	3.07
	Au(100)	–1.31	1.85	3.17
	Au(110)	–1.26	1.78	3.04

**Table 4:** The *E*_pw-AL_, *E*_pw-CL_, and *E*_pw_ of [C_4_mpyrr][Tf_2_N] on Au(*hkl*) at different *j*_cut-off_ values.

*j*_cut-off_/±mA cm^–2^	Crystal face	*E*_pw-CL_/V vs. Fc/Fc^+^	*E*_pw-AL_/V vs. Fc/Fc^+^	*E*_pw_/V
0.1	Au(111)	–3.13	2.26	5.40
	Au(100)	–3.14	2.30	5.44
	Au(110)	–3.13	2.30	5.43

0.5	Au(111)	–3.34	2.40	5.74
	Au(100)	–3.33	2.40	5.73
	Au(110)	–3.36	2.41	5.77

1.0	Au(111)	–3.42	2.46	5.87
	Au(100)	–3.40	2.45	5.85
	Au(110)	–3.43	2.45	5.88

Tables 5 and 6 summarize the *E*_dl_ and *E*_pw_ of [N_1,4,4,4_][Tf_2_N] on Au(*hkl*). The highest *E*_dl_ value for [N_1,4,4,4_][Tf_2_N] was obtained on the Au(100) surface, while that of [N_1,4,4,4_][Tf_2_N] on Au(111) was the lowest. The *E*_pw_ values of the Au(111) and Au(110) surfaces were almost the same at all the *j*_cut-off_ values, whereas the Au(100) surface afforded the widest *E*_pw_. Specifically, when *j*_cut-off_ was ±1.0 mA cm^–2^, the *E*_pw_ was 6.99 V for the reaction on the Au(100) surface, whereas the *E*_pw_ was 6.54 V for that on Au(111), and 6.60 V in the case of the Au(110) surface.

**Table 5:** The *E*_dl-AL_, *E*_dl-CL_, and *E*_dl_ of [N_1,4,4,4_][Tf_2_N] on Au(*hkl*) at different *j*_cut-off_ values.

*j*_cut-off_/±µA cm^–2^	Crystal face	*E*_dl-CL_/V vs. Fc/Fc^+^	*E*_dl-AL_/V vs. Fc/Fc^+^	*E*_dl_/V
5	Au(111)	–0.82	0.49	1.31
	Au(100)	–1.49	0.52	2.01
	Au(110)	–0.80	0.52	1.32

10	Au(111)	–1.55	0.60	2.14
	Au(100)	–1.70	0.63	2.33
	Au(110)	–1.68	0.63	2.31

20	Au(111)	–1.77	0.76	2.53
	Au(100)	–1.98	0.88	2.87
	Au(110)	–1.95	0.80	2.75

**Table 6:** The *E*_pw-AL_, *E*_pw-CL_, and *E*_pw_ of [N_1,4,4,4_][Tf_2_N] on Au(*hkl*) at different *j*_cut-off_ values.

*j*_cut-off_/±mA cm^–2^	Crystal face	*E*_pw-CL_/V vs. Fc/Fc^+^	*E*_pw-AL_/V vs. Fc/Fc^+^	*E*_pw_/V
0.1	Au(111)	–3.28	2.02	5.29
	Au(100)	–3.58	2.10	5.68
	Au(110)	–3.31	2.03	5.34

0.5	Au(111)	–3.85	2.28	6.13
	Au(100)	–3.97	2.41	6.39
	Au(110)	–3.87	2.30	6.17

1.0	Au(111)	–4.05	2.49	6.54
	Au(100)	–4.27	2.72	6.99
	Au(110)	–4.08	2.52	6.60

Tables 7 and 8 list the *E*_dl_ and *E*_pw_ of [C_4_mim][I] on Au(*hkl*). The order of the *E*_dl_ values was dependent on *j*_cut-off_, which was due to the difference in the current density measured during the cathodic process. Similarly, the order of the *E*_pw_ values at *j*_cut-off_ = ±0.1 mA cm^–2^ was affected by the peak current density for reductive desorption of the iodine adlayer. At *j*_cut-off_ = 0.5 or 1.0 mA cm^–2^, *E*_pw_ was influenced solely by the cathodic decomposition of [C_4_mim][I] and the anodic reaction involving the complexation of gold with iodide, resulting in the following order of *E*_pw_: Au(100) < Au(111) < Au(110).

**Table 7:** The *E*_dl-AL_, *E*_dl-CL_, and *E*_dl_ of [C_4_mim][I] on Au(*hkl*) at different *j*_cut-off_ values.

*j*_cut-off_/±µA cm^–2^	Crystal face	*E*_dl-CL_/V vs. Fc/Fc^+^	*E*_dl-AL_/V vs. Fc/Fc^+^	*E*_dl_/V
5	Au(111)	–1.64	–0.55	1.09
	Au(100)	–1.29	–0.53	0.76
	Au(110)	–1.13	–0.48	0.65

10	Au(111)	–1.94	–0.51	1.42
	Au(100)	–2.03	–0.49	1.54
	Au(110)	–1.97	–0.44	1.52

20	Au(111)	–2.03	–0.47	1.56
	Au(100)	–2.09	–0.46	1.63
	Au(110)	–2.03	–0.42	1.61

**Table 8:** The *E*_pw-AL_, *E*_pw-CL_, and *E*_pw_ of [C_4_mim][I] on Au(*hkl*) at different *j*_cut-off_ values.

*j*_cut-off_/±mA cm^–2^	Crystal face	*E*_pw-CL_/V vs. Fc/Fc^+^	*E*_pw-AL_/V vs. Fc/Fc^+^	*E*_pw_/V
0.1	Au(111)	–2.15	–0.41	1.74
	Au(100)	–2.49	–0.41	2.08
	Au(110)	–2.20	–0.40	1.79

0.5	Au(111)	–2.65	–0.26	2.39
	Au(100)	–2.64	–0.26	2.38
	Au(110)	–2.67	–0.25	2.42

1.0	Au(111)	–2.80	–0.13	2.68
	Au(100)	–2.79	–0.14	2.65
	Au(110)	–2.86	–0.11	2.74

Tables 9 and 10 show the *E*_dl_ and *E*_pw_ of [C_6_mim][I] on Au(*hkl*). The difference in the current density measured during the cathodic process affected the order of the *E*_dl_ values at each *j*_cut-off_ value. The lowest value of *E*_pw_ was observed for Au(111), whereas Au(100) and Au(110) exhibited nearly equal values. *E*_pw_ at ±1.0 mA cm^–2^ was not measured because the current density did not reach ±1.0 mA cm^–2^.

**Table 9:** The *E*_dl-AL_, *E*_dl-CL_, and *E*_dl_ of [C_6_mim][I] on Au(*hkl*) at different *j*_cut-off_ values.

*j*_cut-off_/±µA cm^–2^	Crystal face	*E*_dl-CL_/V vs. Fc/Fc^+^	*E*_dl-AL_/V vs. Fc/Fc^+^	*E*_dl_/V
5	Au(111)	–1.24	–0.55	0.68
	Au(100)	–1.30	–0.54	0.76
	Au(110)	–0.78	–0.54	0.23

10	Au(111)	–1.98	–0.52	1.46
	Au(100)	–2.11	–0.51	1.60
	Au(110)	–0.91	–0.51	0.40

20	Au(111)	–2.09	–0.50	1.59
	Au(100)	–2.19	–0.48	1.70
	Au(110)	–2.09	–0.48	1.60

**Table 10:** The *E*_pw-AL_, *E*_pw-CL_, and *E*_pw_ of [C_6_mim][I] on Au(*hkl*) at different *j*_cut-off_ values.

*j*_cut-off_/±mA cm^–2^	Crystal face	*E*_pw-CL_/V vs. Fc/Fc^+^	*E*_pw-AL_/V vs. Fc/Fc^+^	*E*_pw_/V
0.1	Au(111)	–2.61	–0.47	2.14
	Au(100)	–2.61	–0.43	2.17
	Au(110)	–2.63	–0.44	2.18

0.5	Au(111)	–2.82	–0.27	2.55
	Au(100)	–2.94	–0.14	2.80
	Au(110)	–2.95	–0.14	2.81

1.0	Au(111)	ND a	ND a	ND a
	Au(100)	ND a	ND a	ND a
	Au(110)	ND a	ND a	ND a

a Not determined because the decomposition current density did not reach ±1.0 mA cm^–2^.

## 2 Experimental Design, Materials and Methods

[C_4_mim][PF_6_] (Merck, >99.0%), [C_4_mpyrr][Tf_2_N] (Solvionic, 99.9%), [N_1,4,4,4_][Tf_2_N] (IoLiTec, >99%), [C_4_mim][I] (Kanto Chemical Co. Ltd., >99%), and [C_6_mim][I] (Kanto Chemical Co. Ltd., >99%) were used in this study. Detailed information about the water, halide, and alkali metal contents, the presence of other impurities, and the color of each IL is provided in Section I of the Supporting Information in ref. [1]. Following the drying of the ILs in vacuum at approximately 80°C for >6 h, they did not exhibit the cathodic stripping peak of gold oxide originating from a trace amount of water in the ILs [10].

Au(111), Au(100), and Au(110) working electrodes were prepared using Clavilier's method [11]. The area of the working electrode was 0.065 ± 0.005 cm^2^. The working electrodes and Pt wires were annealed in a hydrogen flame and cooled in air for 1 min. Thereafter, they were placed inside the antechamber of a vacuum-type glove box (UN650F, UNICO Corp.), followed by evacuation for >15 min. The antechamber was refilled with Ar gas until the vacuum gauge reached –0.1 bar with respect to the atmospheric pressure, whereupon it was re-evacuated. This refill/evacuation cycle was repeated two times. Subsequently, the pressure of the antechamber was increased to atmospheric pressure using Ar gas. The electrodes were transferred from the antechamber to the main room of the glove box, which was maintained at sufficiently low H_2_O and O_2_ concentrations using a gas recycling purification system (MF-71, UNICO). The working electrodes were contacted with the ILs in three-electrode cells using Pt wires as counter and quasi-reference electrodes.

LSV was conducted while maintaining the contact between the working electrode and IL in a hanging-meniscus configuration. The detailed steps of LSV and data analysis have been explained in “How the data were acquired” and “Description of data collection” in the Specifications Table. *E*_dl_ and *E*_pw_ were calculated using the following equations:

(1) $$E_{\text{dl}}=E_{\text{dl}-\text{AL}}-E_{\text{dl}-\text{CL}}\;\left(j_{\text{cut}-\text{off}}=\pm 5,\;\pm 10,\;\text{or}\;\pm 20\;\mu A\;cm^{-2}\right)$$

(2) $$E_{\text{pw}}=E_{\text{pw}-\text{AL}}-E_{\text{pw}-\text{CL}}\;\left(j_{\text{cut}-\text{off}}=\pm 0.1,\;\pm 0.5,\;\text{or}\;\pm 1.0\;\text{mA}\;cm^{-2}\right)$$

where *E*_dl-AL_ and *E*_pw-AL_ are the electrode potentials at which the positive *j*_cut-off_ values are measured, and *E*_dl-CL_ and *E*_pw-CL_ are the electrode potentials at which the negative *j*_cut-off_ values are measured. The *j*_cut-off_ values for *E*_dl_ and *E*_pw_ were chosen based on previous studies [7,[12], [13], [14], [15]. The potential values of LSV were referenced to the redox potential of 2 mM ferrocene (Fc) in the corresponding IL, as recommended by IUPAC [16]. The Fc/Fc^+^ redox couple has been used widely to characterize ILs [17], [18], [19], [20].

## Ethics Statement

Not applicable.

## CRediT authorship contribution statement

**Hiroyuki Ueda:** Conceptualization, Data curation, Formal analysis, Investigation, Methodology, Validation, Visualization, Writing – original draft, Writing – review & editing. **Soichiro Yoshimoto:** Data curation, Funding acquisition, Project administration, Supervision, Writing – review & editing.

## Declaration of Competing Interest

The authors declare that they have no known competing financial interests or personal relationships that could have appeared to influence the work reported in this paper.
